# Postabortion Care and the Voluntary Family Planning Component: Expanding Contraceptive Choices and Service Options

**DOI:** 10.9745/GHSP-D-19-00128

**Published:** 2019-08-22

**Authors:** Douglas Huber

**Affiliations:** a Reproductive Health Specialist, Newton, MA, USA.

## Abstract

Universal access to voluntary postabortion family planning is a critical and compelling component of postabortion care. Such access should be joined with postpartum family planning services in national programs, health information systems, and training programs. The same providers and facilities deliver both services, and integration could yield cost efficiencies and increased coverage for women receiving postabortion care.

## POSTABORTION FAMILY PLANNING WITHIN POSTABORTION CARE: A COMPELLING MANDATE

Worldwide, 1 in 4 pregnancies ends by induced abortion, and almost half of the 56 million abortions per year are unsafe.^1^ For many women, the lack of voluntary postabortion family planning will soon lead to another induced abortion, given that fertility often returns in 2 to 3 weeks after induced abortion and miscarriage.^2^^–^^4^ Postabortion family planning counseling and services must be accessible to all women who present for postabortion care (PAC) as their right and as a moral and professional obligation of providers and programs. Anything less is negligence.

Strong support for universal access comes from international health associations of obstetricians/gynecologists, midwives, and nurses who are committed to making voluntary postabortion family planning a standard of practice in PAC and to working across their professions to optimize services^2^:

If the woman we treat for postabortion complications is there because she could not get contraception, we have failed her. If she leaves without family planning, we have failed her twice.

Collectively these associations represent more than 10 million doctors, midwives, and nurses providing health care for women in more than 110 countries. Joining them are major donors and policy makers who support health professionals in providing universal access to voluntary postabortion family planning as part of comprehensive maternal health services to achieve the Millennium Development Goals and Family Planning 2020 (FP2020) objectives.^5^

## POSTABORTION FAMILY PLANNING: WHAT WORKS?

The articles in this supplement demonstrate success with postabortion family planning as a component of PAC in a range of settings supported by multiple donors and governments. These articles describe successes in providing postabortion family planning in humanitarian crisis settings, in reaching youth, and by offering postabortion family planning following misoprostol treatment with PAC.^6^^–^^9^ Seeing the diverse range of organizations, countries, and stakeholders sustaining these PAC programs and generating these reports is encouraging.

The authors describe progress in providing a broad range of family planning methods, including long-acting reversible contraceptives (LARCs); highlight new technologies for communication; document the perspectives of patients and providers, including their views on what is still needed to optimize PAC services; and identify cost-effective strategies for governments, programs, and donors.^10^^–^^14^

The authors also document that misunderstanding and misconceptions about contraceptives remain a major stumbling block in providing the family planning component of PAC to clients; that training in counseling and values clarification is much needed; and that engaging men to support family planning for their partners is important and can be challenging.^6^^–^^10^^,^^12^^,^^14^^–^^16^

### Long-Acting Reversible Contraceptives

LARCs—contraceptive implants and intrauterine devices—represent a common postabortion family planning choice among women served in the PAC programs reported in this supplement.^6^^–^^9^^,^^12^ This finding is understandable, given that implant use is rising rapidly across many sub-Saharan African countries and sociodemographic sectors. Women in challenging life circumstances (e.g., humanitarian crisis settings) as well as youth are choosing highly effective methods according to their needs to determine the timing and spacing of subsequent pregnancies.^6^^–^^9^^,^^12^ Contributing to this expansion is increased commodity availability, trained and competent providers committed to broadening method choice, and recognition of the reproductive realities of women.^17^ As the provision of LARCs for postabortion family planning expands, it is essential that voluntarism and informed choice is emphasized and that clients have access to removal services so they may have their implant or intrauterine device removed when they choose. Permanent methods should also be available at a later date, depending on women’s reproductive intentions and desires.

As part of PAC, written information about the woman’s chosen contraceptive method is needed to reinforce counseling messages, to guide correct use, and to address common misconceptions about contraceptives. The woman also needs to have written information about when to seek care should a complication occur after PAC and where to obtain answers for any questions or concerns. Written materials also serve as job aids for busy health providers, helping them provide correct, credible messages to women as well as their partners and communities.

### New and Emerging Strategic Alliances

A promising development is the strategic approach to integrate postpartum family planning with postabortion family planning, as included in the FP2020 initiative to advance postpartum/postabortion family planning as a global movement.^13^^,^^18^ The health and program rationales are sensible and can be synergistic, given that the same staff and facilities provide both postpartum and postabortion care. Training, supervision, supply systems, and information, education, and communication materials, as well as reporting and monitoring, should be strengthened in parallel for postpartum and postabortion family planning while recognizing differences where they exist. Integrating postpartum and postabortion family planning should be a priority for ministries of health, policy makers, training institutions, donors, and NGOs.

Engaging the faith community can advance PAC—and postabortion family planning as a core component—drawing on the growing support for voluntary family planning by interfaith networks, faith-based organizations (FBOs), their health care facilities, and religious leaders.^19^^–^^23^ As stated by one multifaith network^19^:

We recognize the importance of access to information about and services to enable families to plan the timing and spacing of their pregnancies consistent with their faith for family wellbeing …

FBOs are major health service providers in many sub-Saharan African countries, where 85% to 90% of people identify religion as very important in their lives.^19^^,^^20^^,^^23^^,^^24^ Voluntary postabortion family planning to reduce unintended pregnancies and repeat abortions resonates well with faith communities. Faith leaders are influential and a number are now family planning champions.^20^^,^^21^^,^^23^ With accurate information, they help address contraceptive misconceptions and misunderstandings in their communities, an ongoing issue in postpartum and postabortion family planning provision. However, FBOs need technical and financial support for updated PAC training, contraceptive management, and monitoring systems.

### Youth

Youth-friendly PAC services reported in this supplement document high levels of voluntary postabortion family planning that meets the needs of youth.^7^^,^^8^ Programs include competency-based provider training on contraceptive methods, values clarification to address bias and improve client-provider interactions, and ongoing provider mentoring. But such efforts can also be costly and labor intensive, a challenge for scaling up countrywide. This is another area in which joint postpartum/postabortion family planning programming may offer synergies in providing family planning access for youth after birth or abortion.

For most health providers, especially in sub-Saharan Africa, although their professional associations endorse postabortion family planning as a standard of practice in PAC, religion is important in their lives as well as those of their clients. Where tensions exist between providers’ values and the health imperative of postabortion family planning for youth, engaging the faith community can facilitate dialogue and values clarification.

## PLAN FOR LONG-TERM SUCCESS

The authors in this supplement highlight several components needed for sustainable postabortion family planning success, including links with postpartum family planning. To be effective, national programs need to monitor voluntary postabortion family planning counseling and provision in their national health management information systems.^8^^,^^12^^,^^13^ Performance monitoring includes documenting the numbers of PAC patients, those counseled, and the number and percentage of women receiving voluntary contraception by specific method.

Several elements of success stand out in these articles:

- Training providers in family planning counseling and updating their knowledge of contraceptive methods through competency-based training and ongoing mentoring. This need was noted by providers, patients, and program managers.^7^^–^^9^^,^^14^^,^^15^
- Establishing a contraceptive logistics system to ensure a full range of contraceptive options at every site providing PAC.
- Recognizing cost as a major barrier for many women and recommending that PAC services, especially a wide range of contraceptive options, be available for free or at minimal cost.^6^^,^^7^^,^^9^^,^^10^^,^^12^^,^^15^^,^^16^
- Decentralizing PAC services to lower-level facilities and expanding the scope of practice for midlevel providers, especially nurses and midwives, to include PAC.^6^^,^^8^^,^^9^^,^^11^^,^^13^^–^^15^
- Discussing with women their reproductive intentions, rapid return of fertility, potential complications after treatment, and if a contraceptive is desired, essential information about the woman’s chosen contraceptive method.^6^^,^^8^^,^^10^^–^^14^^,^^16^ To supplement counseling on these key points, health professionals call for providing women with user-friendly written information, which also provides a job aid for providers.^2^^–^^5^ Content is readily available from the World Health Organization’s *Family Planning:* A *Global Handbook for Providers*, the accompanying wall chart, and “Facts for Family Planning,” among others.^25^^,^^26^

## CONCLUSION

The articles in this supplement demonstrate how to provide quality PAC services and increase voluntary postabortion contraceptive use, including LARCs, in remote and challenging environments, in youth-friendly services, and in decentralized primary health care centers. Voluntary postabortion family planning initiatives should be unified with postpartum family planning as a global movement for post-pregnancy health care. Postpartum/postabortion family planning expansion promises to be cost efficient and central to women’s health—a compelling rationale for national health programs and no longer an isolated service.

Advocacy and support for these programs is expanding among health professional associations, communities, religious leaders, FBOs, donors, and international health organizations. Now is the time to make voluntary postabortion family planning universally accessible as an essential component PAC.

## Notes

**Funding:** Support was provided by EngenderHealth with funding from the United States Agency for International Development (USAID).
